# Estimating the Double Burden of Malnutrition among 595,975 Children in 65 Low- and Middle-Income Countries: A Meta-Analysis of Demographic and Health Surveys

**DOI:** 10.3390/ijerph16162886

**Published:** 2019-08-13

**Authors:** Blessing J. Akombi, Stanley Chitekwe, Berhe W. Sahle, Andre M.N. Renzaho

**Affiliations:** 1 School of Social Sciences and Psychology, Western Sydney University, Penrith, NSW 2571, Australia; 2 School of Public Health and Community Medicine, University of New South Wales, Sydney, NSW 2052, Australia; 3 United Nations International Children’s Emergency Fund, Lalitpur 44600, Nepal

**Keywords:** stunting, wasting, underweight, overweight, undernutrition, overnutrition, low- and middle-income countries, double burden of malnutrition

## Abstract

Introduction: Given the changing global nutrition landscape, the double burden of malnutrition is a major public health challenge in many developing countries. The main aim of this study is to estimate the double burden of malnutrition among children in low- and middle-income countries (LMICs). Methods: This study used cross-sectional data from Demographic and Health Surveys (2001–2016). A meta-analysis was conducted to estimate the prevalence of malnutrition indicators in 595,975 children under five years from 65 LMICs. Significant heterogeneity was detected among the various surveys (I2 >50%), hence a random-effect model was used. Sensitivity analysis was also performed, to examine the effects of outliers. Results: The pooled estimate for stunting, wasting, underweight, and overweight/obesity was 29.0%, 7.5%, 15.5%, and 5.3% respectively. Countries with the highest coexistence of undernutrition and overweight/obesity were: South Africa (stunting 27.4% (95% CI: 25.1, 29.8); overweight/obesity 13.3% (95% CI: 11.5, 15.2)), Sao Tome and Principe (stunting 29.0% (95% CI: 26.8, 31.4); overweight/obesity 10.5% (95% CI: 9.0, 12.1)), Swaziland (stunting 28.9% (95% CI: 27.3, 30.6); overweight/obesity 10.8% (95% CI: 9.7, 12.0)), Comoros (stunting 30.0% (95% CI: 28.3, 31.8); overweight/obesity 9.3% (95% CI: 8.3, 10.5)), and Equatorial Guinea (stunting 25.9% (95% CI: 23.4, 28.7); overweight/obesity 9.7% (95% CI: 8.0, 11.6)). Conclusions: There is an urgent need to strengthen existing policies on child malnutrition to integrate and scale up opportunities for innovative approaches which address the double burden of malnutrition in children under five years in LMICs.

## 1. Introduction

Child malnutrition in all its forms is a major public health challenge in low- and middle-income countries (LMICs). Malnutrition refers to a lack of proper nutrition either from insufficient nutrient and/or energy intake (undernutrition) or excessive nutrients and/or energy intake (overnutrition). Undernutrition and overnutrition have different effects on the child’s development and cognitive performance. Child undernutrition including stunting, wasting, and underweight adversely affects survival, physical and cognitive development, and reproductive and economic productivity of individuals as well as increasing susceptibility to acute and chronic diseases in adulthood [1,2].

However, childhood undernutrition is closely linked to overweight and obesity later in life. According to the World Health Organisation (WHO), children who have suffered from undernutrition and were born with low birthweight or stunted are at far greater risk of being overweight and obese when faced with energy-dense diets and a sedentary lifestyle later in life [3]. Childhood overnutrition negatively affects a child’s physical health, and social and emotional well-being resulting in low self-esteem, depression, and social isolation as well as obesity-related medical conditions including liver disease, sleep apnea, type 2 diabetes mellitus, cardiovascular disease, and asthma and other respiratory problems [4]. Most LMICs experience the co-existence of both undernutrition and overnutrition in the same population. This phenomenon, popularly known as the “double burden” of malnutrition, is rapidly gaining global attention, especially in LMICs [5,6], hence, the need for action plans that target both undernutrition and overnutrition simultaneously.

The WHO double-duty actions aim to integrate and harness the synergetic effect of multiple interventions, programs, and policies to simultaneously address the burden of both undernutrition and overnutrition as well as diet-related noncommunicable diseases [7]. The approach recommends the adoption of multisectoral approaches combining nutrition-specific (addressing immediate causes of undernutrition) and nutrition-sensitive (addressing underlying causes of undernutrition) interventions to effect a more holistic sustainable response to mitigate the short- and long-term effects of the double burden of undernutrition [8,9].

Therefore, to properly operationalize the WHO double-duty actions and maximize pre-existing policies linked to nutrition in the Sustainable Development Goals (SDGs), baseline data on the double burden of child malnutrition are urgently needed, especially in LMICs. Hence, the aim of this study is to estimate the double burden of malnutrition among children under five years in LMICs to identify high-priority countries for implication of the WHO double-duty actions, thus making proper use of limited resources.

## 2. Materials and Methods

### 2.1. Data Sources

This study used data from the most recent Demographic and Health Survey (DHS) of 65 LMICs. The datasets are publicly available from the DHS website [10]. DHS are population-based and nationally representative surveys with high response rates (>90%). The surveys are comparable across countries and have large sample sizes (usually between 5000 and 30,000 households). The households were selected using a multistage cluster sampling method. Three core questionnaires are used in DHS surveys: A household questionnaire, a women’s questionnaire, and a men’s questionnaire. Eligible participants in all households were women and men aged 15 ± 49 years. Details on data collection and sampling methodology employed by DHS are described elsewhere [10]. Only countries with DHS post-2000 AD were included in the analysis in order to capture the double burden of malnutrition from the introduction of the millennium development goals (MDGs).

### 2.2. Outcome Variable

Child malnutrition was measured by stunting, wasting, underweight, and overweight/obesity in children under five years. The DHS manual [11] indicates that the age of the child was determined from the date of interview and the date of birth of each child. Weight was measured using SECA digital scales and recorded in kilograms (kg) to the nearest 0.1 kg. The height was measured using a wooden support board (Shorr Board) and recorded in centimeter (cm) to the nearest 0.1 cm. Children <2 years or <85 cm were measured lying down (length) and those ≥2 years ≥85 cm were measured standing up (height). Stunting, underweight, and wasting were defined as height-for-age, weight-for-age, and weight-for-height <−2 standard deviations of the 2006 WHO Standard Growth Reference. Overweight/obesity was defined as weight-for-height >2 standard deviations [12]. For countries with DHS data prior to 2006, the anthropometric indicators were recalculated using WHO growth standards [12]. Stunting was used as the key indicator for monitoring child undernutrition as endorsed by the World Health Assembly (WHA) [13].

### 2.3. Statistical Analysis

Analyses were performed using Stata version 14.0 (StataCorp, College Station, TX, USA). The syntax “metaprop” was used to generate forest plots for each of the malnutrition indicators. Each forest plot showed the prevalence of an indicator in individual countries, its associated 95% confidence intervals (CI), and corresponding weight. A test of heterogeneity of the DHS data obtained for the different countries showed a high level of inconsistency (I2 > 50%), hence a random-effect model [14] was used in the meta-analysis. Sensitivity analyses were conducted to examine the effect of outliers by using a method similar to that employed by Patsopoulos and colleagues [15] which involves comparing the pooled prevalence before and after elimination of one country at a time.

## 3. Results

Table 1 shows the malnutrition indicators for 595,975 children under-5 years in 65 LMICs.

### 3.1. Stunting

**Figure 1:**
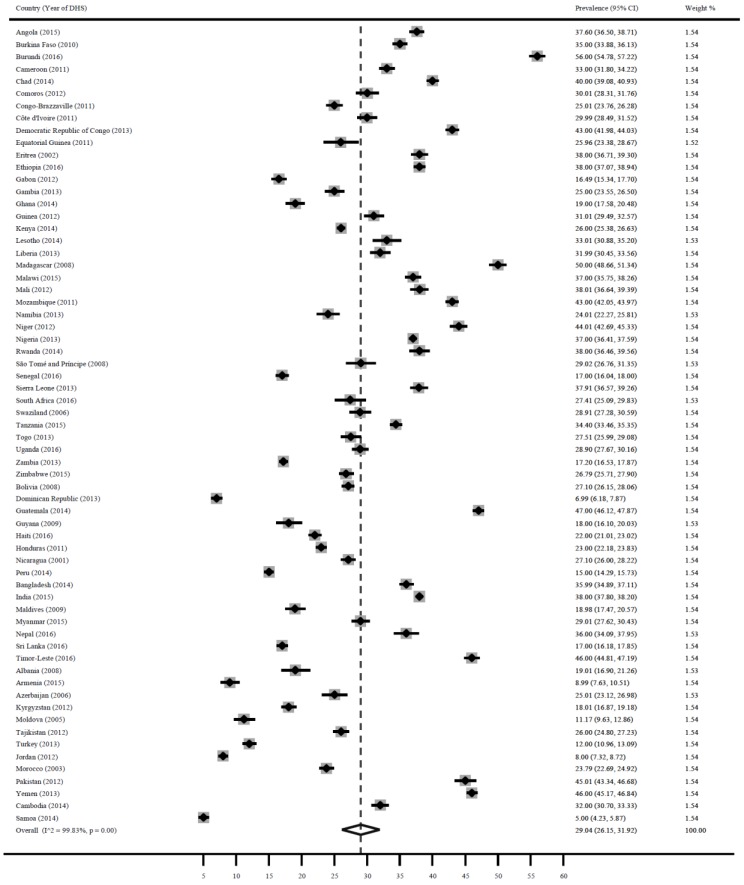
Prevalence of child stunting in LMICs.

Figure 1 shows a forest plot of the prevalence of stunting among children under 5 years in LMICs. The pooled estimate for stunting was 29.0% (95% CI: 26.2, 31.9). Countries with the highest prevalence were: Burundi 56.0% (95% CI: 54.8, 57.2), Madagascar 50.0% (95% CI: 48.7, 51.3), Guatemala 47.0% (95% CI: 46.1, 47.9), Timor-Leste 46.0% (95% CI: 44.8, 47.2), Yemen 46.0% (95% CI: 45.2, 46.8), Pakistan 45.0% (95% CI: 43.3, 46.7), Niger 44.0% (95% CI: 42.7, 45.3), Mozambique 43.0% (95% CI: 42.1, 43.9), Democratic Republic of Congo 43.0% (95% CI: 41.9, 44.0) and Chad 40.0% (95% CI: 39.1, 40.9). While countries with the lowest prevalence were: Samoa 5.0% (95% CI: 4.2, 5.9), Dominican Republic 7.0% (95% CI: 6.2, 7.9), Jordan 8.0% (95% CI: 7.3, 8.7), Moldova 8.0% (95% CI: 6.7, 9.5), Armenia 9.0% (95% CI: 7.6, 10.5).

### 3.2. Wasting

**Figure 2:**
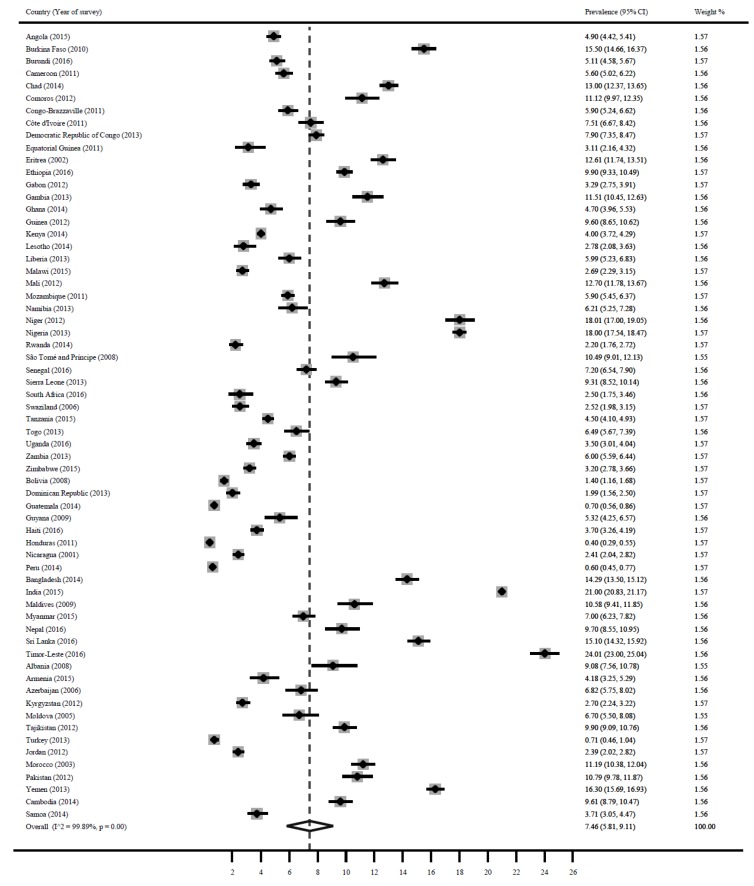
Prevalence of child wasting in LMICs.

Figure 2 shows a forest plot of the prevalence of wasting among children under 5 years in LMICs. The pooled estimate for wasting was 7.5% (95% CI: 5.8, 9.1). Wasting was highest in Timor-Leste 24.0% (95% CI: 23.0, 25.0), India 21.0% (95% CI: 20.1, 21.2), Nigeria 18.0% (95% CI: 17.5, 18.5), Niger 18.0% (95% CI: 17.0, 19.1), Yemen 16.3% (95% CI: 15.7, 16.9), Burkina Faso 15.50% (95% CI: 14.7, 16.4), Sri Lanka 15.1% (95% CI: 14.3, 15.9), Bangladesh 14.3% (95% CI: 13.5, 15.1), Chad 13% (95% CI: 12.4, 13.7) and Eritrea 12.6% (95% CI: 9.9, 12.4). Wasting was lowest in Honduras 0.4% (95% CI: 0.3, 0.6), Peru 0.6% (95% CI: 0.5, 0.8), Guatemala 0.7% (95% CI: 0.6, 0.9), Turkey 0.7% (95% CI: 0.5, 1.0), Bolivia 1.4% (95% CI: 1.2, 1.7), Dominican Republic 2.0% (95% CI: 1.6, 2.5), Kazakhstan 1.8% (95% CI: 0.9, 3.2), and Nicaragua 2.0% (95% CI: 1.7, 2.4).

### 3.3. Underweight

**Figure 3:**
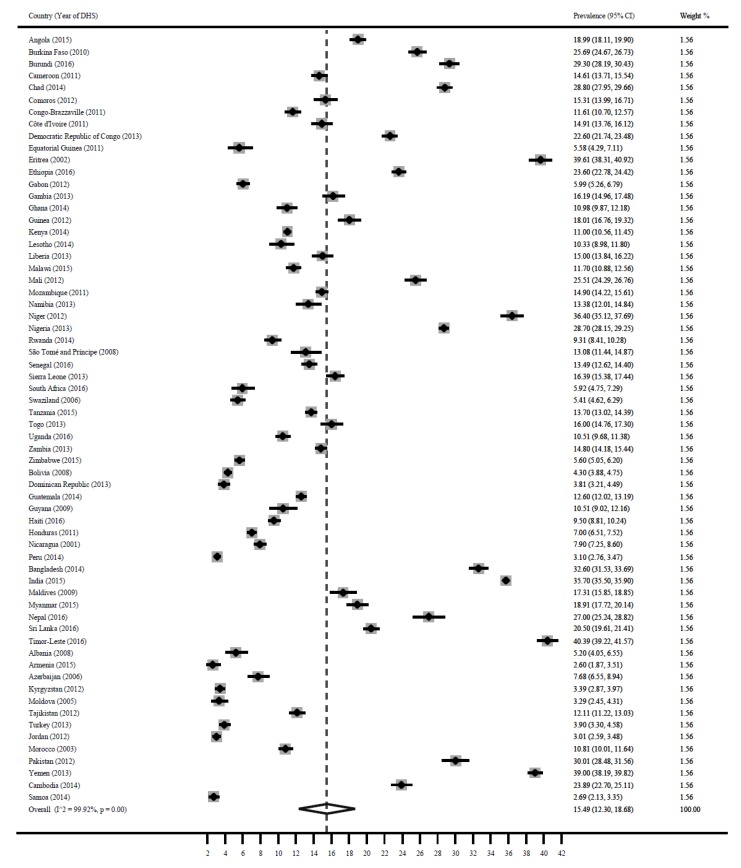
Prevalence of child underweight in LMICs.

Figure 3 shows a forest plot of the prevalence of underweight among children under 5 years in LMICs. The pooled estimate for underweight was 15.5% (95% CI: 12.3, 18.7). Timor-Leste 40.4% (95% CI: 39.2, 41.6), Eritrea 39.6% (95% CI: 38.3%, 40.9), Yemen 39% (95% CI: 38.2, 39.8), Niger 36.4% (95% CI: 35.1, 37.7), India 35.7% (95% CI: 35.5, 35.9), Bangladesh 32.6% (95% CI: 31.5, 33.7), Mauritania 31.8% (95% CI: 30.3, 33.4), Pakistan 30% (95% CI: 28.5, 31.6), Burundi 29.3% (95% CI: 28.2, 30.4) and Nigeria 28.7% (95% CI: 28.2, 29.3) reported the highest prevalence for underweight. Paraguay 0.3% (95% CI: 0.14, 0.54), Armenia 2.6% (95% CI: 1.9, 3.5), Samoa 2.7% (95% CI: 2.1, 3.4), Jordan 3.0% (95% CI: 2.6, 3.5), Peru 3.1% (95% CI: 2.8, 3.5), Kyrgyzstan 3.4% (95% CI: 2.9, 3.9), Dominican Republic 3.8% (95% CI: 3.2, 4.5), Turkey 3.9% (95% CI: 3.3, 4.6) and Bolivia 4.3% (95% CI: 3.9, 4.8) reported the lowest prevalence for underweight.

### 3.4. Overweight/Obesity

**Figure 4:**
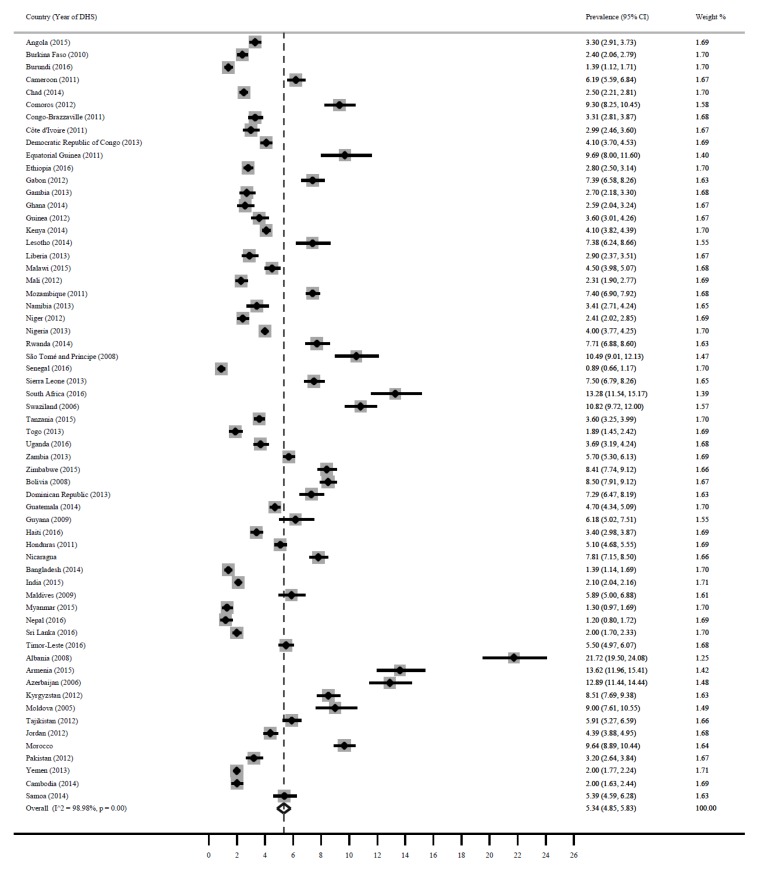
Prevalence of child overweight/obesity in LMICs.

Figure 4 shows a forest plot of the prevalence of overweight/obesity among children under 5 years in LMICs. The pooled estimate for overweight/obesity was 5.3% (95% CI: 4.9, 5.8). Countries with the highest prevalence were: Albania 21.7% (95% CI: 19.5, 24.1), Azerbaijan 12.9% (95% CI: 11.4, 14.1), South Africa 13.3% (95% CI; 11.5, 15.2), Armenia 13.6% (95% CI: 11.9, 15.4), Sao Tomo and Principe 10.5% (95% CI: 9.0, 12.1). While childhood overweight/obesity was lowest in Senegal 0.9% (95% CI: 0.7, 1.2), Nepal 1.2% (95% CI: 0.8, 1.7). Myanmar 1.3% (95% CI: 0.9, 1.7), Burundi 1.4 (95% CI: 1.1, 1.7), Bangladesh 1.4% (95% CI: 1.1, 1.7), and Togo 1.9% (95% CI: 1.5, 2.4).

## 4. Discussion

This study estimated the double burden of child malnutrition in LMICs. Countries with the highest burden of child undernutrition were Timor-Leste, Burundi, Yemen, Nigeria, Madagascar, India, Guatemala, Niger, and Eritrea. Child undernutrition has consequences on morbidity, mortality, human capital development, and economic productivity [16]. Research has shown that most LMICs experience shortage in food supply [17,18,19], environmental challenges such as drought and climate change [20], harmful economic systems, conflict, and limited access to land for agricultural purposes [21] and these could adversely affect agricultural productivity, food security, and child nutrition [22]. Child nutritional status could also be adversely influenced by socio-economic, demographic and agro-ecological factors such as rising cost of living, rapid population growth and desertification [23,24,25], which are prevalent in most LMICs.

Countries with the highest burden of child overweight/obesity were Albania, Azerbaijan, Sao Tomo and Principe, South Africa, and Armenia. The food environment has been a primary driver of the rise in childhood overweight/obesity worldwide over the past half-century [26,27]. Studies have shown that changes in global diets and the global food system due to globalization and trade liberalization could have an adverse effect on nutrition in LMICs [27,28]. Global trade could influence food systems, particularly the availability, accessibility, and affordability of nutritious food, which may lead to the uptake of nutritionally deficient food [27,28].

Countries experiencing the highest coexistence of child undernutrition and overweight/obesity were South Africa, Sao Tome and Principe, Swaziland, Comoros, and Equatorial Guinea.

Optimal child nutrition is a key determinant in achieving global health targets. The growing double burden of malnutrition in LMICs presents a major global public health concern, which, if not properly addressed, might prevent the SDGs from being met. Existing nutrition interventions mostly target single forms of malnutrition [29] and this may lead to slow achievement of the global nutrition target as observed within most LMICs. With limited fiscal and human resources, it becomes necessary to identify opportunities where reduction in multiple forms of malnutrition could be achieved with single interventions. Therefore, policy, programs, and interventions which open effective channels that focus on the simultaneous reduction of both undernutrition and overweight can help accelerate progress in achieving the SDGs. This is the potential of “double-duty actions” [7].

As highlighted in the United Nations Decade of Action on Nutrition 2016–2025 [7], the integrated approach adopted in the double-duty actions leverage on the coexistence of multiple forms of malnutrition and has the potential to address the double burden of child malnutrition via three pathways. First, the “do no harm” approach ensures that current policies, programs and interventions addressing one form of child malnutrition are not inadvertently increasing the risk of other forms of malnutrition. This is mostly observed when interventions targeted at reducing acute child undernutrition through improvement of child feeding practices inadvertently increases a child’s susceptibility to long-term risks of overweight. The primary concern this approach aims to address is the imbalance in the impact of nutrition interventions. Second, the “retrofit” approach which leverages on existing actions addressing single forms of malnutrition to simultaneously influence other forms of malnutrition. For instance, initiatives which target exclusive breastfeeding in the first 6 months of life could also address excessive maternal weight gain in the postpartum period, which in turn protects against obesity and some noncommunicable diseases later in life for both infant and mother [30]. This approach is cost-effective with a huge potential for double returns on existing initiatives. It also provides policymakers and program managers with more efficient and integrated action plans to address multiple forms of malnutrition. Finally, the third approach involves development of de-novo initiatives to proactively address the double burden of malnutrition building from identified shared drivers of different forms of malnutrition. This could involve identifying new initiatives targeting the most significant shared drivers of malnutrition in a particular country. Considering that the type of initiative deployed and its effectiveness will vary between and within countries depending on each country’s unique local epidemiology, policy, cultural, environmental, and food contexts, therefore, to advance the potential of double-duty actions in countries, it is vital to assess existing national policies and evaluate their impact on outcomes associated with both undernutrition and overnutrition.

This study had some strength. First, the DHS are nationally representative and population-based surveys with large sample sizes. Second, the variables used by DHS are comparable across all countries [10]. However, this study also had some limitations. First, some LMICs were not included because they either had no DHS data or had no comprehensive data on the malnutrition indicators or their DHS was post-2000 AD. Second, the estimation of child nutritional status was limited to weight and height, with limited data on more detailed components of nutritional status, such as body composition or biochemical or metabolic status. Third, due to the difference in years of survey, the results are not comparable for present conditions in some nations.

## 5. Conclusions

The burden of child malnutrition varies within LMICs; therefore, interventions to address the double burden of malnutrition should be appropriate to the country setting. Such interventions should simultaneously target shared drivers of both undernutrition and overweight from shared platforms. Additionally, there is an urgent need to assess existing policies and their alignments with the Sustainable Development Goals 2 (Zero Hunger) and 3 (Good Health and Well-Being), and to evaluate impact, dissemination strategies, and scaling up opportunities of innovative approaches to prevent the double burden of child malnutrition in LMICs.

## Figures and Tables

**Table 1:** Low- and middle-income Countries and their malnutrition indicators for children under 5 years.

Country	DHS	No. of Children (N)	No. of Stunted Children	No. of Wasted Children	No. of Underweight Children	No. of Overweight/Obese Children
Angola	2015	7455	2803	365	1416	246
Burkina Faso	2010	6994	2448	1084	1797	168
Burundi	2016	6464	3620	330	1894	90
Cameroon	2011	5860	1934	328	856	363
Chad	2014	10,854	4342	1411	3126	271
Comoros	2012	2762	829	307	423	257
Congo-Brazzaville	2011	4591	1148	271	533	152
Côte d’Ivoire	2011	3581	1074	269	534	107
DRC *	2013	9030	3883	713	2041	370
Equatorial Guinea	2011	1094	284	34	61	106
Eritrea	2002	5466	2077	689	2165	No data
Ethiopia	2016	10,447	3970	1034	2465	293
Gabon	2012	3856	636	127	231	285
Gambia	2013	3372	843	388	546	91
Ghana	2014	2895	550	136	318	75
Guinea	2012	3531	1095	339	636	127
Kenya	2014	18,986	4936	759	2088	778
Lesotho	2014	1869	617	52	193	138
Liberia	2013	3520	1126	211	528	102
Madagascar	2008	5436	2718	No data	No data	No data
Malawi	2015	5752	2128	155	673	259
Mali	2012	4857	1846	617	1239	112
Mozambique	2011	10,313	4435	608	1537	763
Namibia	2013	2287	549	142	306	78
Niger	2012	5481	2412	987	1995	132
Nigeria	2013	26,190	9690	4714	7517	1048
Rwanda	2014	3813	1449	84	355	294
São Tomé and Príncipe	2008	1544	448	162	202	162
Senegal	2016	5722	973	412	772	51
Sierra Leone	2013	5094	1931	474	835	382
South Africa	2016	1401	384	35	83	186
Swaziland	2006	2940	850	74	159	318
Tanzania	2015	9848	3388	443	1349	355
Togo	2013	3282	903	213	525	62
Uganda	2016	5148	1488	180	541	190
Zambia	2013	12,328	2120	740	1825	703
Zimbabwe	2015	6352	1702	203	356	534
Bolivia	2008	8422	2282	118	362	716
Dominican Republic	2013	3619	253	72	138	264
Guatemala	2014	12,567	5906	88	1583	591
Guyana	2009	1522	274	81	160	94
Haiti	2016	6618	1456	245	629	225
Honduras	2011	10,167	2338	41	712	519
Nicaragua	2001	6277	1701	151	496	490
Peru	2014	9540	1431	57	296	No data
Bangladesh	2014	7318	2634	1046	2386	102
India	2015	219,796	83,522	46,157	78,467	4616
Maldives	2009	2513	477	266	435	148
Myanmar	2015	4088	1186	286	773	53
Nepal	2016	2422	872	235	654	29
Sri Lanka	2016	7865	1337	1188	1612	157
Timor-Leste	2016	6798	3127	1632	2746	374
Albania	2008	1289	245	117	67	280
Armenia	2015	1579	142	66	41	215
Azerbaijan	2006	1979	495	135	152	255
Kyrgyzstan	2012	4337	781	117	147	369
Moldova	2005	1522	170	102	50	137
Tajikistan	2012	5080	1321	503	615	300
Turkey	2013	3668	440	26	143	No data
Jordan	2012	5851	468	140	176	257
Morocco	2003	5682	1352	636	614	548
Pakistan	2012	3466	1560	374	1040	111
Yemen	2013	13,823	6359	2253	5391	276
Cambodia	2014	4893	1566	470	1169	98
Samoa	2014	2859	143	106	77	154

* DRC means Democratic Republic of Congo.
